# Disparity of anemia prevalence and associated factors among rural to urban migrant and the local children under two years old: a population based cross-sectional study in Pinghu, China

**DOI:** 10.1186/1471-2458-14-601

**Published:** 2014-06-14

**Authors:** Shiyun Hu, Hui Tan, Aiping Peng, Hong Jiang, Jianmei Wu, Sufang Guo, Xu Qian

**Affiliations:** 1 School of Public Health, Fudan University, Shanghai, China; 2 Global Health Institute, Fudan University, Shanghai, China; 3 Key Laboratory of Public Health Safety, Ministry of Education, Shanghai, China; 4 Shanghai Maternal and Child Health Center, Shanghai, China; 5 Pinghu Institute of Maternal and Child Health, Zhejiang Province, China; 6 United Nations Children’s Fund China Country Office, Beijing, China

## Abstract

**Background:**

Number of internal rural to urban migrant children in China increased rapidly. The disparity of anemia prevalence among them and children of local permanent residents has been reported, both in big and middle-size cities. There has been no population-based study to explore the associated factors on feeding behaviors in small size cities of China. This study aimed to identify whether there was a difference in the prevalence of anemia between children of rural to urban migrant families and local children under 2 years old in a small coastal city in China, and to identify the associated factors of any observed difference.

**Methods:**

A community-based, cross-sectional survey was conducted in Pinghu, a newly-developing city in Zhejiang Province, China, among the caregivers of 988 children (667 who were identified as children of migrants and 321 locals) aged 6–23 months. Disparity of anemia prevalence were reported. Association between anemia prevalence and socio-economic status and feeding behaviors were explored among two groups respectively.

**Results:**

Anemia prevalence among the migrant and local children was 36.6% and 18.7% respectively (aPR 1.86, 95% CI 1. 40 to 2.47). Results from adjusted Poisson models revealed: having elder sibling/s were found as an associated factor of anemia with the aPR 1.47 (95% CI 1.16 to 1.87) among migrant children and 2.58 (95% CI 1.37 to 4.58) among local ones; anemia status was associated with continued breastfeeding at 6 months (aPR = 1.57, 95% CI 1.15 to 2.14) and lack of iron-rich and/or iron-fortified foods (aPR = 0.68, 95% CI 0.50 to 0.89) among the migrant children but not among local ones.

**Conclusion:**

Anemia was more prevalent among migrant children, especially those aged 6–11 months. Dislike their local counterparts, migrant children were more vulnerable at early life and seemed sensitive to feeding behaviors, such as, over reliance on breastfeeding for nutrition after aged 6 months, lack of iron-rich and/or iron-fortified foods. Future strategies to narrow the gap of anemia prevalence between the migrant and local children should target more susceptible groups and through improvement of feeding practices among younger children in those kinds of newly-developing areas of China.

## Background

Internal and cross-border migration is closely associated with globalization, economic development, urbanization and social-economic disparities. Along with market reform, China has witnessed rapid unbalanced economic growth, not only between urban and rural areas, but also between different regions. The emerging private markets for housing and employment have opened new opportunities for social mobility in China since the 1980s [1]. Millions of rural residents have migrated to urban areas, primarily from the less developed central-western parts to the well-developed eastern coastal region seeking employments opportunities [2,3]. The number of internal rural to urban migrants has increased dramatically in China within last few decades, reaching 221 million (17% of total population in 2011) [4]. This number is anticipated to double within the next 10 years [5] and with a greater impact on small-size cities [6]. Increasingly, migrants move with their family members to the cities in which they work, leading to an increasing number of migrant children residing in those cities [7]. For example, in Zhejiang Province, which holds the second largest internal migrants population in China, there were more than 1.2 million migrant children under 14 years old in 2010, 5 times more than that in 2000 [8].

The process of migration has a mixed influence in children’s health. It may provide opportunities for better access to health care services and a more diversified diet [9]. On the other hand, social factors such as the fundamental change in living environment and a lack of family support may result in poorer health due to changes in feeding practices [10,11]. The unique “*Hukou*” (household registration) system in China also complicates the situation. The *Hukou* determines the social welfare packages such as healthcare, education, employment, and housing, and differs for agricultural or non-agricultural residences [12], which causes difference on social benefits when rural residents with an agricultural *Hukou* move to the cities [13]. Migrant children, therefore, share very low utilization of healthcare in the immigrant cities [14].

Anemia has a high prevalence among young children specifically in Africa and Asia. World Health Organization (WHO) has estimated that, globally, 293 million preschool aged children are anemic and 16.8 million of them live in China. More than 40% of the world’s anemia burden is caused by iron deficiency. Anemia in young children may have a long-term effect on their neurological development and behavior, some of which is irreversible [15]. The disparity of anemia prevalence among children of migrant workers (migrant children) and children of local permanent residents (local children) has been reported, both in big [16,17] and middle-size cities [18,19]. However, there has been no population-based study to explore the associated factors especially on feeding behaviors among young migrant and local children in small size cities of China. In 2011, a project financed by UNICEF was launched to improve the health of migrant children. Based on this project, we reported the results of a survey conducted in a small coastal city in China. This survey aimed to generate evidence for future policy formulation on anemia prevention, by measuring the anemia of migrant and local children aged 6–23 months, and identify associated factors focusing on social-economic status and feeding behaviors.

## Methods

### Study site

This study was conducted in Pinghu, a coastal city in eastern Zhejiang Province, which has a high density of immigrants [20]. 303,000 migrants account for almost 40% of total residents, with a higher proportion of women and children (more than 60% of the city’s children and half of the women of childbearing age) in 2011.

### Study population and sampling

Our study population comprised children aged 6–23 months and their main caregivers. Children were selected with the following inclusion criteria: migrant children where: neither parents’ *Hukou* was in Pinghu and at least one parent living in Pinghu for more than 6 months; and local children where: at least one parent was registered as a Pinghu permanent resident. Children were excluded if they were seriously ill or hospitalized or away from the city. If there were two or more children in one family, only the youngest was included. Age of 6 to 23 months was applied because children in this age range are at highest risk of anemia [15].

All 193 suburbs within 10 communities (still named town or township) served as clusters. In each *Resident Administrative Committee* (the smallest government administration agency) the list of eligible children within their areas was compiled. Information of local children was accessed from the computerized residence registration system and the migrant children mainly from the latest immunization programme and then double-checked with the records from migration administration agencies, family planning management committees and county child health service station.

Multi-stage sampling method was conducted based on the WHO advocated cluster sampling design. The number of samples required per cluster was calculated according to the 2009 WHO Maternal, Newborn and Child Health Household Survey Guidelines [21]. Sampling frames were generated for the migrant and the local groups separately. As a survey with multiple indicators, we assumed the proportion of children with stunting for migrant children to be 15% and local children to be 7%, and prevalence of anemia for migrant children to be 20% and local children to be 10% [22-25]. The study anticipated a design effect of two, and a 90% response rate. Therefore, 25 suburbs were sampled for the migrant children and 25 for the local children, based on probability proportional to size (PPS). Within each selected suburbs, 48 migrant and 24 local children were selected separately by revised random walking method, where the sampling start point was randomly selected from the registered addresses.

### Data collection and quality control

The fieldwork was conducted in local community health centers (CHCs) between October and November, 2011, with a pilot study conducted in 25 households from August 2 to 10, 2011. All participants were interviewed face to face at their nearest CHCs, and the hemoglobin of each child was measured after informed consent. Interviewers (physicians from local MCH institutes and CHCs) and quality controllers (lecturers and senior post-graduate students from School of Public Health, Fudan University) were trained prior to conducting the survey. The survey questionnaires were routinely checked on the field and audited by external quality controllers. Re-interviews either by phone or home visits were conducted if transcription or logical problems were identified in the surveys, or where missing values were found during data cleaning or data analysis stage.

### Measurement

#### ***Dependent variables***

Hemoglobin (Hb) concentration of children was measured by photometer (capillary Hb level (HemoCue® Hb 201+ [HemoCue, Angelholm, Sweden], precision of 1 g/L). About 10 μ/l blood sample of each child was collected from the left ring or middle finger by HemoCue Microcuvettes. Since Pinghu is at sea level, altitude adjustment was skipped. Children with hemoglobin level <110 g/L (WHO recommendation [26]) were defined as anemia.

#### ***Independent variables***

A structured questionnaire was designed for primary caregivers, who were mostly the mothers. The questionnaire consisted of socio-demographic information (children’s date of birth and gender), background of mother (education, occupation, type of *Hukou*) and family (annual income and family structure), child health care received and practices on feeding (including breastfeeding).

Age of children was coded into trisections (‘6 months~’, ‘12 months~’ and ‘18 ~ 23 months’). Education of mother was recoded into either ‘low’ (illiteracy, primary school); ‘medium’ (junior middle school); or, ‘high’ (senior middle school or higher), based on five categories of education levels in the questionnaire. Occupation of mother was recoded into “employed” or “not work for payment” based on a question with five occupational categories. One household whose members cooked and eat together was defined as one family. Annual family income was collected and categorized by P25 (RMB 50,000 Yuan) and P75 (RMB 100,000 Yuan) (percentile) of annual family income of local permanent residents (<P25 as poor, P25 to P75 as middle and > P75 as rich). Number of family member and relatives’ appellation were collected and coded into two indicators. Family structure was recoded into parents only or living not only with parents. This indicates the caregivers of children. Number of children was recoded into one-child family and family had more than one child that indicates number of children that the family needs to look after.

Child health care (growth and development monitoring and nutrition counseling) received each year after birth was collected. Timely child health care was defined in accordance with China National Child Health Care Protocol version 2011 (>3 times by aged 6–7 months; >4 times by aged 8–11 months; >5 times by aged 12–17 months; >6 times by aged 18–23 months).

Details on feeding practices were collected based on a 24-hour dietary recall (WHO recommendation: the initiation of breastfeeding module and infant and young child feeding (IYCF) module) [27]. Four binominal variables for feeding practices including breastfeeding at 6 months, consumption of iron-rich and/or iron-fortified foods, minimum dietary diversity (MDD) and minimum meal frequency (MMF) were calculated as recommended by WHO [27]. Breastfeeding at 6 months: for children aged 6–23 months continually fed breast milk after aged 6 months. Consumption of iron-rich and/or iron-fortified foods means proportion of children aged 6–23 months who receive an iron-rich food or iron-fortified foods that is specially designed for infants and young children, or that is fortified in the home. Iron-rich foods was define as flesh foods, including any organ meats such as liver, kidney, heart, or others; any meat, such as beef, pork, lamb, goat, chicken, or duck; fresh or dried fish, shellfish or seafood. Iron-fortified foods include commercially fortified foods specially designed for infants and young children which contain iron, or foods fortified in the home with a micronutrient powder containing iron or a lipid-based nutrient supplement containing iron. MDD defined as proportion of children aged 6–23 months who receive foods from 4 or more food groups. (Among a total of 7 food groups: dairy products, legumes and nuts, flesh foods, eggs, vitamin A rich fruits and vegetables, cereals and tubers, and other fruits and vegetables). MMF is proportion of breastfed and non-breastfed children 6–23 months of age who receive solid, semi-solid, or soft foods (but also including milk feeds for non-breastfed children) the minimum number of times or more. For breastfed children, the minimum number of times varies with age (two times if aged 6–8 months and three times if aged 9–23 months). For non-breastfed children, the minimum number of times does not vary by age (four times for all children aged 6–23 months).

### Statistical analysis

Table 1 summarizes the definition and coding of variable used. Descriptive analysis was conducted to present anemia prevalence among young migrant and local children through Chi square test. Poisson regression and Cochran’s Mantel-Haenszel (CMH) test were used to compare the anemia prevalence between migrant and local children aged 6–23 months. Crude Prevalence Ratios between potential risk factors and outcomes were first calculated by using Poisson regression. Adjusted Poisson regression models were then developed to assess the association between social-economic status, feeding behaviors and anemia. Either significant variables (Crude Prevalence Ratios > 1) or variables were identified as risk factors of childhood anemia in the literature [28-30] were retained. Three multiple Poisson regression models were developed and performed respectively among migrant and local children. Model 1 was used to explore the association between anemia and social-economic status including age and sex of children; education level and occupation of mother; annual family income, family structure (caregiver/s) and number of children (with or without sibling/s). For the feeding behaviors, breastfeeding at 6 months and iron intake could possibly be the proxy of the two other feeding indicators. Therefore two models developed respectively to assess the association between breastfeeding at 6 months and iron intake (model 2), or MDD and MMF (model 3) with anemia. Prevalence Ratios (PR) was calculated adjusted for timely child health care and all factors in model 1. No interactions between the breastfeeding at 6 months and iron intake or MDD and MMF in both groups were found. Due to ongoing economic development and population mobilization, mother’s Hukou (agricultural and nonagricultural) couldn’t distinctly reflect the differences, such as urban/rural dwelling and economic status, of two types of people. As we have adjusted for education and job status as well as feeding practices, we decide not to analyse the association of the outcome with type of mother’s Hukou. Crude Prevalence Ratios (cPR) and adjusted Prevalence Ratios (aPR) with 95% CI were reported. STATA for windows version 10.1 (Stata Corporation, College Station) was used for statistical analysis.

**Table 1:** Definition of variables used in the analysis

**Variable**	**Values**		
***Dependent variables***			
Anemia (hemoglobin level <110 g/L)	1. Yes	0. No	
***Independent variables***			
Age of child (months)	1. 6 ~ 11	2. 12 ~ 17	3. 18 ~ 23
Gender of child	1. Boy	0. Girl	
Education of mother	1. Low	2. Medium	3. High
Occupation of mother	1. Not work for payment	0. Employed	
Annual family income (thousand RMB Yuan)	1. Poor < 50	2. Middle 50-100	3. Rich > 100
Caregivers	1. Parents only	0. Parents & others	
Elder sibling/s^a^	1. Yes	0. No	
Timely child health care^b^	1. Yes	0. No	
Breastfeeding at 6 months^c^	1. Yes	0. No	
Iron^d^	1. Yes	0. No	
MDD^e^	1. Yes	0. No	
MMF^f^	1. Yes	0. No	

^a^Elder sibling/s: family had more than one child indicate the investigated child at least has one elder sibling/s.

^b^Timely child health care: child health care received by age(month) in time.

^c^Breastfeeding at 6 months: for children aged 6–23 months fed breast milk at aged 6 months.

^d^Iron: consumption of iron-rich and/or iron-fortified foods.

^e^MDD: minimum dietary diversity.

^f^MMF: minimum meal frequency.

### Ethics considerations

The study protocol and informed consent procedures were approved by the Ethics Committee (IRB), School of Public Health, Fudan University. Informed consent was acquired from each main caregiver of the children.

## Results

### General characteristics

A total of 988 children (667 migrant and 321 local) and their caregivers were investigated. Characteristics of the study population are presented in Table 2. There were distinct socio-demographic differences between the two populations (migrant and local). The age distribution of the two groups was consistent with the sample frame. There were more young children in the migrant group while the local children were evenly distributed in each age group. Both groups had slightly more boys; however, no statistic significance was found.

**Table 2:** Characteristics and related practices of study population (N = 988)

		**Total**	**Migrant**	**Permanent**	P*
		**% (N = 988)**	**% (n = 667)**	**% (n = 321)**	
**Children**					
Age	6 ~	41.4 (409)	44.7 (298)	34.6 (111)	0.009
(Months)	12~	30.4 (300)	29.1 (194)	33.0 (106)	
	18~	28.2 (279)	26.2 (175)	32.4 (104)	
Gender	Boy	55.6 (549)	56.2 (375)	54.2 (174)	0.550
	Girl	44.4 (439)	43.8 (292)	45.8 (147)	
**Mother**					
Education	Low	19.1 (189)	26.2 (175)	4.4 ( 14)	<0.001
	Medium	55.9 (552)	62.9 (419)	41.4 (133)	
	High	25.0 (247)	10.9 ( 73)	54.2 (174)	
Occupation	Employed	55.6 (549)	45.0 (300)	77.6 (249)	<0.001
	Not work for payment	44.4 (439)	55.0 (367)	22.4 ( 72)	
**Family**					
Annual income	Poor	48.1 (475)	59.8 (399)	23.7 ( 76)	<0.001
	Middle	34.5 (341)	28.5 (190)	47.0 (151)	
	Rich	17.4 (172)	11.7 ( 78)	29.3 ( 94)	
Caregivers	Parents only	40.6 (401)	55.8 (372)	9.0 ( 29)	<0.001
	Parents & others	59.4 (587)	44.2 (295)	91.0 (292)	
Elder sibling/s	Yes	30.2 (298)	37.2 (248)	15.6 ( 50)	<0.001
	No	69.8 (690)	62.8 (419)	84.4 (271)	
**Child health care & feeding**					
Timely child health care	Yes	11.0 (109)	5.2 ( 35)	23.1 (74)	<0.001
	No	89.0 (879)	94.8 (632)	84.4 (247)	
Breastfeeding at 6 months	Yes	74.4 (735)	78.4 (523)	66.0 (212)	<0.001
	No	25.6 (253)	21.6 (144)	34.0 (109)	
Iron	Yes	88.0 (869)	83.5 (557)	97.2 (312)	<0.001
	No	12.0 (119)	16.5 (110)	2.8 ( 9)	
MDD	Yes	77.3 (764)	72.1 (481)	88.2 (283)	<0.001
	No	22.7 (224)	27.9 (186)	11.8 ( 38)	
MMF	Yes	77.8 (769)	73.5 (490)	86.9 (279)	<0.001
	No	22.2 (219)	26.5 (177)	13.1 ( 42)	

^*^Pearson Chi-square test.

Migrant mothers had low education comparing with local mothers (26.2% vs. 4.4%). Less migrant mothers were employed compared with the local mothers and consequently family income level was significantly lower in migrant families. More migrant children (55.8%) lived only with parents, versus 9.0% for the local (P < 0.001). More migrant children had elder siblings than local children (37.2% vs. 15.6%, P < 0.001). Less migrant children had received timely growth monitor and nutrition counseling, much lower than the local children (5.2% vs. 23.1%, P < 0.001). Compared to their local counterparts, more migrant children were breastfeed at 6 months (78.4% vs. 66.0%, P < 0.001). For other feeding practices, less migrant children were provided with iron-rich and/or iron-fortified foods (83.5% vs. 97.2%, P < 0.001), or had achieved minimum dietary diversity (72.1% vs. 88.2%, P < 0.001) and minimum meal frequency (73.5% vs. 86.9%, P < 0.001) on the previous day of the survey.

### Prevalence of anemia

**Figure 1:**
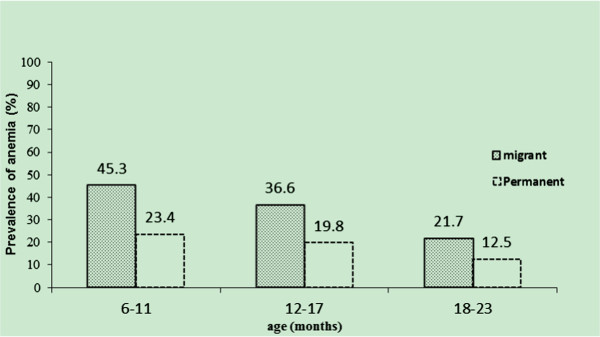
**Anemia prevalence among migrant and local children aged 6–23 months.** (Cochran’s Mantel-Haenszel statistics (CMH) P < 0.001).

Table 3 revealed that 244 (36.6%) migrant children and 60 (18.7%) of the local children were diagnosed as anemic. Adjusted for age and gender of children, migrant children were found to be 1.86 times more likely to have anemia compared with their local counterparts (95% CI 1.40 to 2.47). This disparity remained within almost each age group at statistically significant levels. Within both migrant and local groups younger children were more likely to have anemia. At the same time, younger migrant children had higher risk of anemia prevalence compared with local children and the risks decline as children getting older as aPR = 1.93, (95% CI 1.27 to 2.94) among children aged 6–11 months, aPR = 1.85, (95% CI 1.14 to 3.01) among 12–17 months and aPR = 1.78 (95% CI 0.95 to 3.35) among 18–23 months respectively (Figure 1).

**Table 3:** Comparison of anemia status between migrant and local children aged 6–23 months*

**Age(month)**	**Migrant**		**Local**		**cPR**	**aPR(95% CI)**^**a**^
	**n/N**	**%**	**n/N**	**%**		
6-11	135/298	45.3	26/111	23.4	1.93	1.93(1.27, 2.94)
12-17	71/194	36.6	21/106	19.8	1.85	1.85(1.14, 3.01)
18-23	38/175	21.7	13/104	12.5	1.74	1.78(0.95, 3.35)
Total	244/667	36.6	60/321	18.7	1.96	1.86(1.40, 2.47)

*Crude and adjusted prevalence ratio (cPR) were estimated by using Poisson regression.

^a^Adjusted for age and gender of child.

### Associated factors

Table 4 presented the anemia prevalence and potential associated factors in migrant and local children. The 6–11 months and 12–17 months age groups had a higher anemia prevalence compared with the 18–23 months group among the migrant. Anemia was more prevalent among migrant children with breastfeeding at 6 months, didn’t consume iron-rich and/or iron fortified foods during previous day. Local children whose mothers were not working for payment and who were from family with more than one child were more likely to be anemic.

**Table 4:** Prevalence of anemia in migrant and local children of Pinghu China^*^

	**Migrant(N = 667)**		**Local(N = 321)**	
	**Prevalence of anemia, %(n/N)**	**cPR (95% CI)**	**Prevalence of anemia, %(n/N)**	**cPR (95% CI)**
Age of child (months)				
6~	45.3 (135/298)	**2.09(1.46, 2.99)**	23.4 (26/111)	1.87(0.96, 3.65)
12~	36.6 (71/194)	**1.69(1.14, 2.50)**	19.8 (21/106)	1.58(0.79, 3.17)
18 ~ 23	21.7 (38/175)	1.0	12.5 (13/104)	1.0
Gender of child				
Boy	36.0 (135/375)	0.96(0.75, 1.24)	17.8 (31/174)	0.90(0.54, 1.50)
Girl	37.3 (109/292)	1.0	19.7 (29/147)	1.0
Education of mother				
Low	37.1 (65/175)	0.93(0.60, 1.45)	14.3 (2/14)	1.37(0.32, 5.77)
Medium	35.8 (150/419)	0.90(0.61, 1.34)	19.5 (26/133)	1.29(0.31, 5.37)
High	39.7 (29/73)	1.0	18.4 (32/174)	1.0
Occupation of mother				
Not work for pay	40.6 (149/367)	1.28(0.99, 1.66)	27.8 (20/72)	**1.73(1.01, 2.96)**
Employed	31.7 (95/300)	1.0	16.1 (40/249)	1.0
Annual family income				
Poor	39.1 (156/399)	1.16(0.87, 1.55)	25.0 (19/76)	1.38(0.72, 2.66)
Middle	33.7 (64/190)	0.91(0.57, 1.46)	15.9 (24/151)	0.88(0.47, 1.64)
Rich	30.8 (24/78)	1.0	18.1 (17/94)	1.0
Caregivers				
Parents only	37.1 (138/372)	1.03(0.80, 1.33)	13.8 (4/29)	0.72(0.26, 1.98)
Parents & others	35.9 (106/295)	1.0	19.2 (56/292)	1.0
Elder sibling/s				
Yes	41.1 (102/248)	1.21(0.94, 1.57)	36.0 (18/50)	**2.32(1.34, 4.03)**
No	33.9 (142/419)	1.0	15.5 (42/271)	1.0
Timely child health care				
Yes	22.9 (8/35)	0.61(0.30, 1.24)	18.9 (14/74)	1.02(0.56, 1.85)
No	37.3 (236/632)	1.0	18.6 (46/247)	1.0
Breastfeeding at 6 months				
Yes	40.0(209/523)	**1.64(1.15, 2.35)**	21.7(46/212)	1.69(0.93, 3.07)
No	24.3(35/144)	1.0	12.8(14/109)	1.0
Iron				
Yes	32.3(180/557)	**0.56(0.42, 0.74)**	18.9(59/312)	1.70(0.24, 12.28)
No	58.2(64/110)	1.0	11.1(1/9)	1.0
MDD				
Yes	33.9 (163/481)	0.78(0.60, 1.02)	18.7(53/283)	1.02(0.46, 2.24)
No	43.5 (81/186)	1.0	18.4(7/38)	1.0
MMF				
Yes	34.3(168/490)	0.80(0.61, 1.05)	16.1(45/279)	0.45(0.25, 0.81)
No	42.9(76/177)	1.0	35.7(15/42)	1.0

*Estimated by using Poisson regression, Crude Prevalence Ratios (cPR) and its 95% CI presented.

Table 5 presented the analysis of association between social-economic status, feeding behaviors and anemia prevalence among migrant and local children respectively by using three adjusted multiple Poisson regression models. In model 1, in both migrant and local groups, having elder sibling/s were found to be postitively associated with anemia, along with the aPR 1.47 (95% CI 1.16 to 1.87) among migrant children and 2.58 (95% CI 1.37 to 4.85) among local ones. In model 2, continued breastfeeding for children aged 6 months (aPR 1.57, 95% CI 1.152 to 2.14) were positively and consumption of iron-rich and/or iron fortified foods (aPR 0.68, 95% CI 0.50 to 0.89) negatively associated with anemia among migrant children. In model 3, no statistically significant association between MDD, MMF and anemia among both migrant and local children were found.

**Table 5:** Feeding factors associated with anemia in migrant and local children of Pinghu China*

		**Migrant (N = 667)**			**Local (N = 321)**		
		**Model 1**^**a**^	**Model 2**^**b**^	**Model 3**^**c**^	**Model 1**^**a**^	**Model 2**^**b**^	**Model 3**^**c**^
Age of child (months)	6~	**2.03(1.48, 2.80)**	1.92(1.37, 2.69)	1.89(1.34, 2.67)	1.76(0.90, 3.46)	2.05(0.94, 4.44)	1.89(0.85, 4.19)
	12~	**1.69(1.20, 2.39)**	1.72(1.21, 2.44)	1.66(1.17, 2.36)	1.52(0.76, 3.04)	1.63(0.78, 3.40)	1.68(0.80, 3.49)
	18 ~ 23	1.0	1.0	1.0	1.0	1.0	1.0
Gender of child	Boy	0.94(0.75, 1.18)	0.93(0.74, 1.16)	0.95(0.76, 1.19)	0.93(0.55, 1.57)	0.96(0.57, 1.62)	0.92(0.55, 1.56)
	Girl	1.0	1.0	1.0	1.0	1.0	1.0
Education of mother	Low	0.96(0.65, 1.43)	0.96(0.65, 1.42)	0.95(0.64, 1.40)	0.55(0.12, 2.63)	0.64(0.13, 3.10)	0.69(0.14, 3.34)
	Medium	1.05(0.77, 1.42)	1.08(0.79, 1.47)	1.00(0.74, 1.38)	0.78(0.43, 1.43)	0.78(0.42, 1.44)	0.75(0.41, 1.37)
	High	1.0	1.0	1.0	1.0	1.0	1.0
Occupation of mother	Not work for pay	**1.32(1.03, 1.70)**	1.24(0.96, 1.59)	1.31(1.02, 1.68)	1.55(0.89, 2.72)	1.50(0.85, 2.65)	1.52(0.87, 2.68)
	Employed	1.0	1.0	1.0	1.0	1.0	1.0
Annual family income	Poor	1.35(0.94, 1.95)	1.28(0.88, 1.85)	1.34(0.93, 1.94)	1.29(0.64, 2.59)	1.26(0.62, 2.54)	1.23(0.61, 2.47)
	Middle	1.05(0.72, 1.53)	1.02(0.77, 1.28)	1.05(0.72, 1.52)	0.79(0.42, 1.48)	0.77(0.40, 1.46)	0.75(0.39, 1.41)
	Rich	1.0	1.0	1.0	1.0	1.0	1.0
Caregivers	Parents only	1.02(0.79, 1.32)	0.99(0.77, 1.28)	1.00(0.77, 1.29)	0.75(0.26, 2.17)	0.71(0.24, 2.09)	0.71(0.25, 2.05)
	Parents & others	1.0	1.0	1.0	1.0	1.0	1.0
Elder sibling/s	Yes	**1.47(1.16, 1.87)**	1.40(1.10, 1.78)	1.47(1.15, 1.86)	**2.58(1.37, 4.85)**	2.60(1.37, 4.95)	2.47(1.30, 4.68)
	No	1.0	1.0	1.0	1.0	1.0	1.0
Timely child health care	Yes		1.00(0.63, 1.59)	1.00(0.63, 1.59)		1.44(0.72, 2.88)	1.51(0.75, 3.03)
	No		1.0	1.0		1.0	1.0
Breastfeeding at 6 months	Yes		**1.57(1.15, 2.14)**			1.58(0.86, 2.91)	
	No		1.0			1.0	
Iron	Yes		**0.68(0.50, 0.89)**			1.78(0.23, 13.54)	
	No		1.0			1.0	
MDD	Yes			0.87(0.67, 1.13)			1.12(0.49, 2.57)
	No			1.0			1.0
MMF	Yes			0.85(0.65, 1.10)			0.54(0.28, 1.02)
	No			1.0			1.0

*Estimated by using multiple Poisson regression, adjusted Prevalence Ratios (aPR) and its 95% CI presented.

^a^Model 1, the association between age and sex of children; education level and occupation of mother; annual family income, family structure (caregiver/s), number of children (with or without sibling/s) and timely child health care were analyzed.

^b^Model 2, the association between breastfeeding at 6 months, iron intake and anemia were analyzed, adjusted for age and sex of children; education level and occupation of mother; annual family income, family structure (caregiver/s), number of children (with or without sibling/s) and timely child health care.

^c^Model 3, the association between MDD, MMF and anemia were analyzed, adjusted for age and sex of children; education level and occupation of mother; annual family income, family structure (caregiver/s), number of children (with or without sibling/s) and timely child health care.

## Discussion

Based on field survey, we reported the results of a population-based cross-sectional study conducted in Pinghu, an eastern coastal city in China. Comparison of the anemia prevalence and associated factors between migrant children and local children aged 6–23 months had been presented.

Overall anemia prevalence in Pinghu was reported as higher than our assumption, which was based on other researches in China [23-25]. Anemia prevalence among migrant and local children in our study was found to be moderate and mild according to WHO classification [26]. Migrant children had higher anemia prevalence than the local children amongst all age groups. The disparity corresponded to other reports from some big cities such as Shenzhen (9.5% vs. 7.7%) [17] and Xiamen (13.1% vs. 7.3%) [18]. Overall the disparity of anemia prevalence among migrant and local children in Pinghu (36.6% vs. 18.7%) was higher and the disparity was larger than other findings. Moreover, the anemia prevalence among migrant children was similar with the level in rural areas of middle and western China (32% to 46%) [23,31-33]. This could partly be explained by the fact that most migrants in Pinghu were originally from middle and western of China, therefore maintain a similar susceptibility pattern. It also suggests that the process of acculturation of the migrants didn’t significantly influence the prevalence of anemia among their children. Another possible explanation could be, as a recent urbanization area, Pinghu was still relatively under-developed areas compared with big cities like Shenzhen and Xiamen. Health services and health of local people lagged behind the industrial development. The development status of Pinghu City, however, was likely to be representative of other urban settlements transformed from traditional rural areas in current China. Recently the central government of China had launched a nutritional improvement campaign (through delivery of micro-nutrients packages) in western China [34]. But the needs of those children from western part of China who had left for urban life (commonly in coastal or eastern part of the country) had been under covered by public services in their destination of migration. Our findings suggested that anemia among young children in newly-developing areas still deserves higher attention, especially among migrant children at early life. Both migrant and local children had a higher likelihood of anemia in the 6–11 age groups. This corresponded with the findings that children around 6 months were more vulnerable to anemia [15], especially among migrants, probably due to the limited iron blood reserves at birth.

Environmental factors, especially socio-economic status and feeding practices play leading roles in determining nutritional status in the early years of life. The association between anemia prevalence among young children and social economic status of their families may have multiple pathways [30]. One pathway could be low social economic status associated with antenatal anemia. It then contributes to low birth weight and prematurity, both of which increase the risk of childhood anemia [35]. But socioeconomic status (SES), including mother’s education and occupation status, or family income identified as determinants anemia in previous studies [31], did not appear to be significant in our study. No association was observed between children’s anemia and maternal schooling in the present study, confirming the findings of Hadlers et al. in Brazil [36]. One possible reason was socioeconomic factors affect health by means of more proximate (intermediate) determinants such as feeding practices which played dominant role to occurrence of childhood anemic other than SES status. In our study, child with sibling/s in both migrant and local group were more likely to be anemic. This associated factor had been previous reported by Yang et al. in China [33,36] which needed further study to explore the reasons.

Accumulating evidences demonstrate that anemia is a common clinical manifestation of micronutrient deficiency, particularly iron, zinc, vitamin A, vitamin B_12_, vitamin B_2_, folic acid and magnesium [37-42]. Iron deficiency anemia (IDA) is considered the leading contribution to the global burden of anemia [43]. Introduction of complementary food at early age has prominent effect on iron deficiency correction [30,44,45]. These evidences could partly explain the contribution of the influence of food consumption and bioavailability to the IDA. In our study, dietary factors were one of the more significant associated factors to be considered. On one aspect, prolonged exclusively breastfeeding, such as over 6 months old, was reported to be predictor of infant anemia in developing countries [46]. The worsen complementary feeding such as not reaching minimum dietary diversity and minimum meal frequency, lower intake of iron-rich and/or iron-fortified foods especially among migrant children could contribute to low iron blood level among young children which caused higher anemia prevalence among the group. We found the migrant child who was breastfed at aged 6 months and lack of iron supplements was significant associated with anemia prevalence. These two associated factors were not significant in local children. Higher proportion of breastfeeding and lower of complementary diversity hinted there might be a later introduction and lower quality of complementary foods in migrant children. For local children, iron supplement probably not played important role as it to migrant children. Instead, other micronutrient factors, such as Vitamin B_2_ intake [38] probably were more important. All these suggested that breastfeeding and lack of iron supplements were not necessarily increasing the risk of childhood anemia unless over reliance on it for nutrition after aged 6 months.

A few study limitations existed. First, the survey was only based on one city, Pinghu. In order to obtain a clearer picture, future researches should be expanded into other areas, considering the variations across geographic regions. Second, the sample size of local children was defined by the expected anemia prevalence in migrant children in our multiple-indicator study. It might decrease the test effect of the multiple regression analysis. Third, direct epidemiology inference for causality could not be determined due to the cross-sectional design. Bias might also exist, for instance, hemoglobin values measured in capillary samples are higher than in venous, potentially leading to false-negative results [43].

## Conclusions

In conclusion, this study demonstrated anemia prevalence was relatively high among children aged 6–23 months in Pinghu, an eastern coastal city of China. The migrant children were almost twice as likely to be anemic than the local children. Our study also hinted migrant and local children could share same associated factors (with elder siblings) and had their distinct associated factors (continued breastfeeding at aged 6 months and lower intake of iron-rich and/or iron-fortified foods among migrant children but not among local children). Disadvantageous socio-demographic characteristics and inappropriate feeding practices were highly associated with anemia in migrant and local children. Public policies aimed to narrow the nutrition gap between the migrant and local children should target more susceptible groups and through improvement of feeding practices among younger children in those kind of newly-developing areas of China.

## Competing interests

The authors declare that they have no competing interests.

## Authors’ contributions

XQ and SFG conceived and XQ, SYH and SFG designed the study. SYH and XQ developed the field methodology and directed the field work. APP, HJ and JMW helped the data collection and conducted quality control in the field. SYH, HT and HJ analyzed the data. SYH and HT drafted the manuscript. SFG offered valued revision comments. All authors read and approved the final manuscript.

## Authors’ information

Hui Tan is co-first author.

## Pre-publication history

The pre-publication history for this paper can be accessed here:

http://www.biomedcentral.com/1471-2458/14/601/prepub
